# P-1221. Benchmark Efficacy for Humanized Cefiderocol Plasma and Pulmonary Epithelial Lining Fluid (ELF) Exposures Against Gram Negative Isolates in Standardized Murine Lung Infection Model

**DOI:** 10.1093/ofid/ofae631.1403

**Published:** 2025-01-29

**Authors:** Andrew J Fratoni, Alissa Padgett, Erin Duffy, David P Nicolau

**Affiliations:** Hartford Hospital, Hartford, Connecticut; Hartford Hospital, Hartford, Connecticut; CARB-X, Boston, Massachusetts; Hartford Hospital, Hartford, Connecticut

## Abstract

**Background:**

The collaboration for prevention and treatment of MDR bacterial infections (COMBINE) consortium have developed a standardized murine neutropenic lung infection model to align fundamental model elements to reduce inter-laboratory variability. We developed human simulated regimens (HSRs) based on both plasma and ELF exposures for cefiderocol in the COMBINE murine model and herein provide benchmark efficacy against a collection of *Klebsiella pneumoniae* and *Pseudomonas aeruginosa* isolates.

**Table 1.**
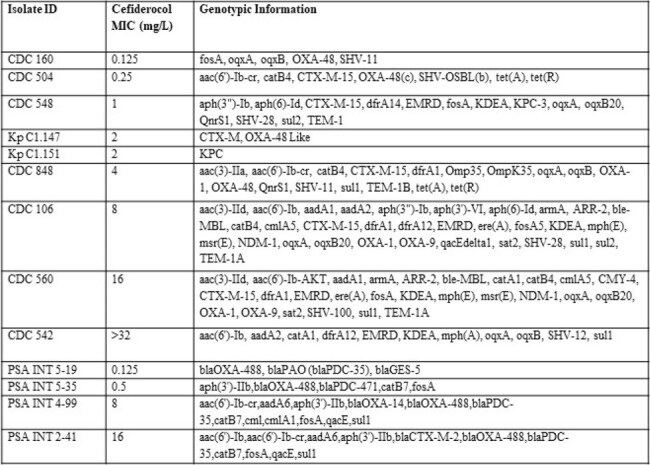
Phenotypic and genotypic information of Klebsiella pneumoniae and Pseudomonas aeruginosa isolates

**Methods:**

Following the COMBINE protocol for the preclinical murine lung model, neutropenic mice that received uranyl nitrate on day -3 to provide predictable renal impairment were administered 0.05 mL intranasal inoculums of *K. pneumoniae* (n=9) or *P. aeruginosa* (n=4) under isoflurane anesthesia. Isolate MICs were determined via BMD. Six mice per isolate were sacrificed 2h after inoculation to determine baseline bacterial burden, and 4-6 received saline control for 24h. Humanized exposures of cefiderocol 2g q8h as 3h infusion in plasma (5, 7.5, 10, 3.5, and 1 mg/kg at 0, 1, 2, 4, and 6h, every 8h) and ELF (3.75, 5, 6.25, 1.75, and 1 mg/kg at 0, 1, 2, 4, and 6h, every 8h) were administered to groups of 6 mice. Efficacy was measured in log_10_ CFU/lung at 24h compared with baseline bacterial burden.

**Figure 1.**
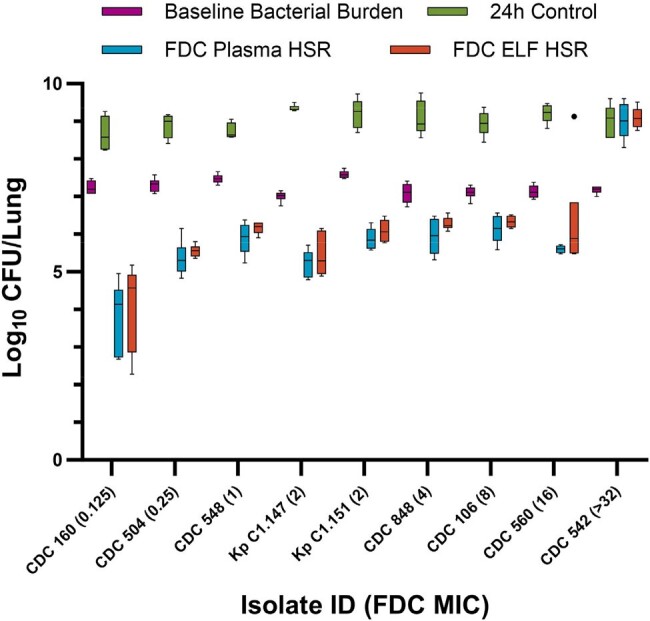
CFU/Lung data in Klebsiella pneumoniae isolates following administration of humanized cefiderocol (2g q8h 3h infusion) plasma and ELF exposures in the COMBINE murine neutropenic lung infection model. Outlier mice determined by Tukey’s Test and displayed as individual dots.

**Results:**

Cefiderocol MICs and genotypic information are presented in Table 1. The CFU/lung data for *K. pneumoniae* and *P. aeruginosa* isolates are displayed in Figures 1 and 2, respectively. The *K. pneumoniae* isolates required higher log_10_ CFU/lung starting bacterial burden (mean ± SD, 7.23 ± 0.18) compared with *P. aeruginosa* (5.68 ± 0.24) to achieve adequate (≥ 1 log_10_ CFU/lung) growth in 24h controls. With the exception of PSA INT 4-99, the FDC plasma HSR, which over-exposes murine ELF relative to human, achieved greater average CFU reduction than the ELF HSR. Differences in CFU reduction between the matrix HSRs were most pronounced in the 2 isolates with MIC of 16 mg/L at the resistance breakpoint.

**Figure 2.**
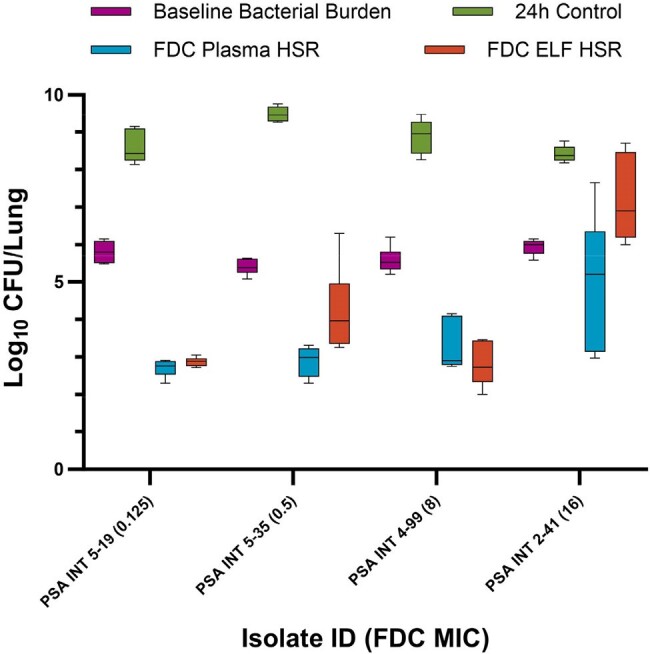
CFU/Lung data in Pseudomonas aeruginosa isolates following administration of humanized cefiderocol (2g q8h 3h infusion) plasma and ELF exposures in the COMBINE murine neutropenic lung infection model.

**Conclusion:**

These data provide a comparative benchmark for expected efficacy with humanized cefiderocol exposures of both plasma and ELF when using the COMBINE protocol. Humanizing exposures at the target site of infection may provide better clinical translation when interspecies differences in penetration from plasma exist.

**Disclosures:**

**Andrew J. Fratoni, PharmD**, InsightRX: Grant/Research Support **Erin Duffy, PhD**, CARB-X: Employee **David P. Nicolau, PharmD**, CARB-X: Grant/Research Support|Innoviva: Grant/Research Support|Innoviva: Honoraria|Merck: Advisor/Consultant|Merck: Grant/Research Support|Merck: Honoraria|Pfizer: Advisor/Consultant|Pfizer: Grant/Research Support|Pfizer: Honoraria|Shionogi: Advisor/Consultant|Shionogi: Grant/Research Support|Shionogi: Honoraria|Venatorx: Grant/Research Support

